# Application of endocrine biomarkers to update information on reproductive physiology in gray whale (*Eschrichtius robustus*)

**DOI:** 10.1371/journal.pone.0255368

**Published:** 2021-08-03

**Authors:** Valentina Melica, Shannon Atkinson, John Calambokidis, Aimée Lang, Jonathan Scordino, Franz Mueter

**Affiliations:** 1 Fisheries Department, College of Fisheries and Ocean Sciences, University of Alaska Fairbanks, Juneau, Alaska, United States of America; 2 Cascadia Research, Olympia, Washington, United States of America; 3 Ocean Associates Inc., on Contract to NOAA Southwest Fisheries Science Center, La Jolla, California, United States of America; 4 Marine Mammal Program, Makah Fisheries Management, Neah Bay, Washington, United States of America; University of Minnesota, UNITED STATES

## Abstract

Most of our knowledge on reproductive biology of gray whales dates back to scientific research conducted during commercial whaling in the late 1950s and 1960s. The goal of the present study was to provide updated insights on reproductive physiology of gray whales, using progesterone and testosterone as biomarkers. We measured hormone concentrations using enzyme immunoassay (EIA) techniques in blubber biopsies collected from 106 individual whales from March to November over a span of 12 years (2004–2016) between California and Alaska. We found testosterone concentrations in males to increase significantly with age (*P* = 0.03). Adult males showed significantly elevated testosterone concentrations when sampled in the fall compared to the summer (*P* = 0.01), likely indicating physiological preparation for mating. We measured testosterone concentrations in females of different age classes, but no statistical differences were found. We found significantly higher progesterone concentrations in pregnant females compared to non-pregnant females and adult males (*P*< 0.001), indicating progesterone is a valid biomarker for pregnancy in gray whales. Both female and male calves had elevated progesterone concentrations, suggesting maternal transfer via lactation. We fit a mixture of two normal distributions to progesterone data from all non-calf females to identify clusters of high and low progesterone and estimated the probability of being pregnant for whales of unknown reproductive status. With this approach we identified likely pregnant and non-pregnant animals. This study represents an important milestone on reproductive profiles in this population, that can be used to estimate more accurate and precise reproductive parameters to be used for better understanding population dynamics of gray whales.

## Introduction

Gray whales *(Eschrichtius robustus)* occur exclusively in the Pacific Ocean [1]. Two populations are recognized, the Western North Pacific (WNP) and Eastern North Pacific (ENP) populations, although some whales that feed in the WNP are known to overwinter in the ENP [2–4]. Both populations were driven close to extinction by commercial whaling, and to date, the WNP population is considered Endangered by the International Union for the Conservation of Nature. The ENP population was removed from the United States endangered species list in 1994 [5] and the most up to date population estimate is 20,580 individuals [6].

The majority of ENP gray whales migrate annually between wintering nursing grounds located in the lagoons and coastal waters of the Baja California Peninsula, Mexico, and feeding grounds located on the continental shelves of the Bering and Chukchi Seas [5]. Among them, a small group of approximately a dozen whales is known to detour their spring migratory route to the North Puget Sound (NPS) to feed on ghost shrimps [7], before continuing northward. A more distinguished ENP subgroup is noted as the Pacific Coast Feeding Group (PCFG), a group of gray whales that terminate their northbound migration further south to forage primarily between southeastern Alaska and northern California from spring to fall [8]. Photo-identification studies of the PCFG started in the 1970s [9–13], with more detailed knowledge acquired over the last couple of decades. According to the definition of the International Whaling Commission’s Scientific Committee [14], PCFG gray whales are those that are seen in the waters between northern California and British Columbia (41°N-52°N; excluding whales observed in Puget Sound) in more than one year between June 1^st^ and November 30^th^. However, photo-ID matches have shown that the feeding habitat of some PCFG whales extends further north, with whales frequently present around Kodiak Island, Alaska [8, 14]. The results of nuclear DNA analyses *inter alia* indicate that PCFG whales interbreed with the ENP whales that feed further north [15, 16], and the PCFG is not considered a separate stock in the United States. Both genetic (based on mitochondrial DNA) and photo-identification studies indicate that matrilineal fidelity to the PCFG occurs [8, 16–18] and the most recent abundance estimate is 232 PCFG whales [8].

Knowledge on the reproductive biology of gray whales is extensive, though outdated as much of it is based on scientific research conducted during commercial whaling off the coast of central California between 1959 and 1969 [19]. The mean age of sexual maturity (ASM) for females and males is estimated at 8 years old (with a range from 5 to 11 years) based on studies of earplug growth layers and gonads [19, 20]. This species has on average a 2-year reproductive cycle [21], with a gestation period of 13 months and calves weaned 6–7 months postpartum [19]. The gray whale migration is staggered in time based on age and reproductive state [19, 22]. For instance, during the southward migration from the feeding grounds, pregnant females migrate first, followed by females that have recently ovulated and adult males, and then by immature whales [1]. Non-pregnant females ovulate during the months of November and December [19], suggesting that mating occurs during the southbound migration [21]. Wintering grounds known for this population include the coastal waters and lagoons on the west coast of the Baja California Peninsula with calving areas in the Laguna Ojo de Liebre (Scammon’s Lagoon) and Laguna San Ignacio [19, 23]. Although some calves are born during the southbound migration [24], most females give birth in the winter grounds by late December or early January [19]. By late January the first phase of northward migration has begun, led by newly pregnant females followed by adult males and juveniles [5]. The second phase of migration occurs in April through May and consists primarily of lactating females with their calves. By summer, the vast majority of gray whales are on their feeding grounds [13, 19].

Over the past two decades, endocrine techniques have been successfully applied to understand and gain information on reproductive processes and parameters for several whale species [25–31]. Nevertheless, the amount of research on gray whale endocrinology is still limited, with few recent studies validating steroid and thyroid hormones in blubber, feces and baleen [32–34]. The use of blubber tissue for endocrine studies has been proven valid and valuable to provide information on reproductive physiology in many odontocete [35–37] and mysticete species [25, 27–29, 31, 38, 39]. Collection of blubber is a minimally invasive technique [40] and over the past two decades, large numbers of skin and blubber samples have been archived in freezers, and subsampled for various types of research (e.g., hormones [27, 29, 41], contaminants [42–45], stable isotopes [38, 46, 47], lipid profiles [48, 49] and age determination [50]). While the perfusion rate of hormones from blood to blubber likely differs among species, blubber concentrations are likely representative of relatively recent (hours to weeks) physiological events [35, 36, 39]. Published studies indicate hormone concentrations in blubber of bowhead whales *(Eubalaena mysticetus)* to reflect those in blood over a time period of weeks [39], whereas in bottlenose dolphins *(Tursiops truncatus)* this lag-time is much shorter (hours) [35, 36]. Sampling efforts to collect blubber biopsies of gray whales have been carried out over the past 25 years along the Pacific West Coast [16, 51, 52].

Sex-steroid hormones such as testosterone and progesterone can be used as biomarkers for reproduction. These steroid hormones are synthesized from cholesterol, mainly in the gonads, and, because of their lipophilic nature, they can be detected and have increasingly been measured in blubber tissues of cetaceans [25, 29, 31, 53, 54]. During pregnancy, progesterone is the predominant sex-steroid hormone and concentrations are elevated [37, 55]. Researchers have used progesterone concentrations in blubber to detect pregnancy in minke whales *(Balaenoptera acurostrata)* [25], bowhead whales [39], humpback whales *(Megaptera novaeangliae)* [27, 38], fin whales *(Balaenoptera physalus)* [31] and blue whales *(Balaenoptera musculus)* [29, 54]. Testosterone is secreted by the gonads and the adrenal glands, and it is the main androgen in mammals [56, 57]. Besides stimulating spermatogenesis, testosterone is responsible for the onset of sexual maturity and involved in the development of both primary and secondary sexual characteristics [37]. As mysticetes are seasonal breeders, testosterone concentrations are likely to have a cyclic trend, peaking before mating, then decreasing after breeding has occurred [58]. Annual cyclicity in testosterone was observed in baleen plates of bowhead, right *(Eubalaena glacialis)* and possibly blue whales [59], and in the blubber of fin [31], blue [54] and humpback whales [41, 60]. Specifically, these studies found testosterone concentrations to be indicative of physiological preparation for reproduction, as they were higher during the time between winter breeding and summer feeding in fin, blue and humpback whales [31, 54, 60].

The present study validates and measures testosterone and progesterone in blubber biopsies of gray whales sampled over a 12-year period, between California and southeastern Alaska, with most of the individuals considered part of the PCFG. The specific research questions were:

- Can progesterone and testosterone be validated and measured in blubber of gray whales?
- Is progesterone an indicator of pregnancy in female gray whales and can it be used to estimate reproductive status for unknown whales?
- Do progesterone and testosterone show variation in response to the age of the individual, time of year and geographic location of sampling?

## Methods

### Sample collection and sighting history

For the present study, we accessed archived biopsy samples (*n* = 119) of gray whale blubber stored frozen at -80° at NOAA Fisheries Southwest Fisheries Science Center (SWFSC) Marine Mammal and Sea Turtle Research Collection. The fieldwork efforts to collect these samples were carried out over a span of 12 years (2004–2016) between March and November by the Marine Mammal Program of the Makah Tribe, Cascadia Research Collective (CRC) and NOAA Fisheries SWFSC [16, 51, 52] (MMPA Permit # 16111–00 and 14097).

The sample set included 106 unique individuals, for which biopsies were matched to photo-identified whales. We identified samples collected from the same individual by comparison of photographs and genetic profiles (see Lang et al. [16] for details on the genetic comparisons). To ensure independence, we included in the main analyses for testosterone and progesterone only one sample per individual, choosing the sample for which age class or reproductive state could be determined. If neither age class nor reproductive state was available or the repeated samples had the same classification, we included the biopsy collected first. Of the 106 unique identified individuals, 89 were whales from the PCFG, two are known to be part of the North Puget Sound (NPS) gray whale group and the remaining 15 were considered part of the overall ENP population.

**Fig 1:**
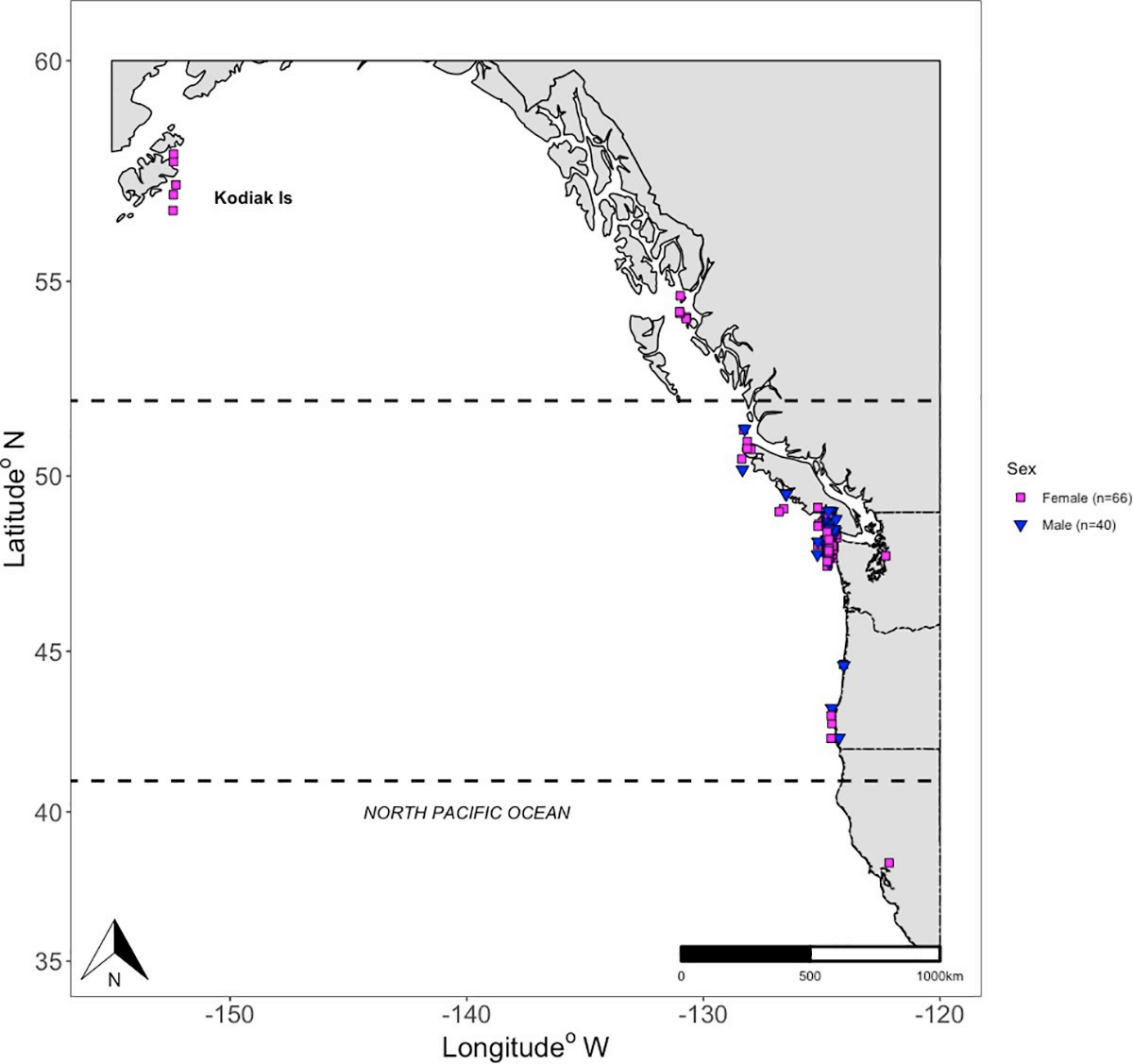
Map of the Pacific Ocean and west coast of North America with gray whales sampling locations color-coded by sex. Blubber biopsy samples were collected off the United States coasts of California (CA, *n* = 2), Oregon (OR, *n* = 6), Washington (WA, *n* = 71) and Alaska (AK, *n* = 5), and in Canadian waters of British Columbia (BC, *n* = 22). Dashed lines mark distribution boundaries of PCFG.

The area of sampling extended as far south as Bodega Bay, CA (Latitude: 38.28°N, Longitude: -122.15°W) north to Kodiak Island, AK (Latitude: 57.36°N, Longitude: -152.42°W) with the majority collected in the IWC-defined PCFG range (Latitude: 41°N– 52°N; [14]). Most of the samples were collected between the months of June and October, thus representing summer and fall, when the whales were on their feeding grounds. One sample was collected in November and three between March and May, two of these were from the same individual. Given the limited sample size, the current study only investigated differences between summer and fall, with summer defined as mid-June to mid-September and fall as mid-September to mid-November. Sampling locations were grouped as California (CA, *n* = 2), Oregon (OR, *n* = 6), Washington (WA, *n* = 71), British Columbia (BC, *n* = 22) and Alaska (AK, *n* = 5) (Fig 1).

The sex of all samples was genetically determined as described in Lang et al. [16] and we accessed the CRC gray whale catalogue to gather life history information, such as year of birth or first sighting, and reproductive status at the time of sampling. We assigned age class and reproductive status at the time of sampling by applying the general criteria defined in similar studies [29–31, 54, 61], with a few modifications. Briefly, we assigned age class based on length of sighting history (LSH) as a proxy for minimum age or based on known age for individuals which were first seen as calves. We identified as calves or young of the year all whales that were small in size (estimated to be less than or equal to 8 meters in length) and accompanied by an adult female [19, 62, 63]. Mean age of sexual maturity for gray whales is estimated to be 8 years, based on histological examinations of gonads and lamina of earplugs [19, 20], therefore we categorized all individuals, males and females, with at least 8 years of LSH or known age as adults. Conversely, we considered all animals of known age less than 8 years as immature (Tables 1 and 2). We further sorted female gray whales by the following reproductive states: calves if sampled as such, immature if they had a known age less than 8 years old when sampled, pregnant if sighted with a calf the year after sampling and lactating if sighted in close association with a small whale noted as calf when sampled (Table 2). We considered as non-pregnant all females from the calf, immature and lactating groups. We classified as adult unknown females with at least 8 years of LSH but with no known reproductive state, and as unknown all females that could not be categorized in any of the age class (i.e., not first identified as calves and with LSH < 8 yrs) or reproductive state categories. Out of the 40 unique male individuals, we identified four calves, six immatures, 16 adults and 14 whales of unknown age class (Table 1), while the female dataset (*n* = 66) was comprised of four calves, six immatures, 36 adults and 20 unknown whales (Table 2). Sighting history (LSH, age class and reproductive status) and sampling information (area, season) as well as hormone concentrations for each whale are summarized in S1 Table.

**Table 1:** Mean (range) of progesterone and testosterone concentrations in male gray whales. Divided by age class.

Age class	Description	Mean testosterone concentrations (range) ng/g	Mean progesterone concentrations (range) ng/g
Calf	Males sighted as calves the year of sampling	0.4 (0.3–0.5)	2.6 (1.8–4.0)
		(*n* = 4)	(*n* = 4)
Immature	Males with known year of birth and known age of less than 8 years	0.4 (0.1–0.9)	0.6
		(*n* = 6)	(*n* = 1)
Adult	Males known to be 8 years of age or more	1.9 (0.2–9.8)	0.5 (0.3–0.6)
		(*n* = 16)	(*n* = 4)
Unknown	Males that do not fit in any other categories	0.6 (0.1–2.6)	NA
		(*n* = 14)	

**Table 2:** Mean (range) of progesterone and testosterone concentrations (ng/g) in female gray whales divided by age class and reproductive state.

Age class description	Reproductive state	Mean progesterone concentrations (range) ng/g	Mean testosterone concentrations (range) ng/g
**Calf**	**Calf**	4.7 (1.5–11.0)	0.8 (0.4–1.2)
Whales sighted as calves the year of sampling		(*n* = 4)	(*n* = 3)
**Immature**	**Immature**	2.2 (0.7–3.5)	0.8 (0.1–2.1)
Whales with known year of birth and known age of less than 8 years		(*n* = 6)	(*n* = 6)
**Adult**	**Lactating**: observed with calf the year of sampling	2.1 (1.5–3.6)	0.4 (0.1–0–8)
Whales known to be 8 or more years of age			
		(*n* = 6)	(*n* = 3)
	**Pregnant:** observed with a calf the year after sampling	19.5 (11.2–30.8)	0.2 (0.2–0.3)
		(*n* = 4)	(*n* = 4)
	**Adult-unknown**: not seen or seen not accompanied by calf	7.9 (0.7–48.9)	0.2 (0.1–0.4)
		(*n* = 26)	(*n* = 2)
**Unknown**	**Unknown**	9.2 (0.6–61.7)	NA
Females that do not fit in any other categories		(*n* = 20)	

Given the unique opportunity to evaluate trends in hormone concentrations in multiple samples from the same animals, we analyzed repeated samples separately. Repeated samples were collected from 8 females and 5 males of different age classes or reproductive states, at different times of the year and were used to test seasonal differences in hormone concentrations (Table 3). Because of the limited sample size, we combined calves and juveniles in one age class group (calf/juvenile) for statistical analysis.

**Table 3:** Minimum age, reproductive state, date of sampling, season, area, and progesterone or testosterone concentrations in repeated samples collected from eight females and five males.

**FEMALES (*n* = 8)**						
**CRC ID**	**Min Age**	**Age class (reproductive state)**	**Date of sampling**	**Season**	**Area**	**Progesterone (ng/g)**
**92**	19	adult (pregnant)	8/6/12	Summer	WA	14.8
	22	adult (unknown)	9/30/15	Fall	BC	10.7
**196**	14	adult (unknown)	7/29/10	Summer	WA	1.6
	19	adult (pregnant)	10/27/15	Fall	BC	11.2
**525**	10	adult (unknown)	9/14/10	Summer	WA	0.2
	15	adult (lactating)	10/6/15	Fall	BC	2.0
**826**	6	unknown (unknown)	9/20/10	Summer	WA	0.8
	11	adult (unknown)	10/20/15	Fall	BC	2.4
**860**	1	calf/juvenile (immature)	9/23/04	Fall	WA	6.0
	9	adult (unknown)	8/1/12	Summer	WA	1.5
**1053**	0	unknown (unknown)	10/30/08	Fall	WA	19.7
	5	unknown (unknown)	6/29/13	Summer	WA	2.4
**1172**	3	unknown (unknown)	8/6/12	Summer	WA	2.2
	6	unknown (unknown)	10/5/15	Fall	WA	0.7
**1512**	1	calf/juvenile (immature)	10/4/13	Fall	WA	2.
	2	calf/juvenile (immature)	6/23/14	Summer	WA	6.0
**MALES (*n* = 5)**						
**CRC**	**LSH**	**Age class**	**Date of sampling**	**Season**	**Area**	**Testosterone (ng/g)**
**510**	15	adult	10/27/15	Fall	BC	10.0
	10	adult	9/19/10	Summer	CA	0.3
**714**	13	adult	10/29/15	Fall	OR	2.7
	9	adult	9/20/11	Summer	WA	0.2
**1303**	4	calf/juvenile	9/30/15	Fall	BC	0.2
	3	calf/juvenile	7/15/14	Summer	WA	0.2
**1604**	2	unknown	9/30/15	Fall	BC	0.4
	0	unknown	8/21/13	Summer	WA	1.0
**1693**	0	calf/juvenile	6/22/13	Summer	WA	0.5
	2	calf/juvenile	10/18/15	Fall	BC	1.5

LSH, length of sighting history; BC, British Columbia; WA, Washington; CA, California; OR, Oregon.

### Assay validation and steroid hormones measurements

For hormones extractions, we cut blubber biopsies by length. Masses of the subsamples weighed between 0.03 and 0.19 g (mean (±SD) = 0.11 (±0.03) ng/g) and were placed into 12 × 75 mm borosilicate glass tubes. We followed the extraction protocol reported in Atkinson et al. [29] and Melica et al. [54], modified from the methods by Mansour et al. [25], and Kellar et al. [53]. Briefly, we manually macerated each blubber sample in 500 μl ethanol using a Teflon tissue homogenizer and centrifuged at 3,000 rpm for 20 min. We poured the supernatant off into a clean tube and repeated this step. We added the supernatant from the second extraction to the supernatant from the first extraction and dried it under forced air. To the dried extract, we added an aliquot of 2 ml of ethanol:acetone (4:1), vortexed, and centrifuged for 15 min. We then poured the supernatant into a new glass tube and dried it under forced air. To each tube we added one milliliter of diethyl ether, vortexed, centrifuged for 15 min, transferred to clean glass tubes, and dried under forced air. For the final extraction, we added 1 ml of acetonitrile to each residue and vortexed, then we added 1 ml of hexane and vortexed. We centrifuged the samples for 15 min, recovered the acetonitrile and extracted it again with additional 1 ml of hexane. At this final step, we removed the hexane layer, and dried the acetonitrile residue under forced air. Extracts were stored frozen at -20°C until assayed.

We measured testosterone and progesterone concentrations using enzyme immunoassay (EIA) techniques (Arbor Assay Kits, Ann Arbor, MI, K032-testosterone and K025-progesterone), which were read with a plate reader Tecan INFINITE 200M NANO. For EIA analysis, we rehydrated extracts with 1 ml of methanol and aliquoted based on the dilution required for each assay. Specifically, we transferred into clean tubes 125 μl of methanol from each extract tested for testosterone, and between 15 and 125 μl from samples tested for progesterone. After drying the methanol aliquots under forced air, we added 125–150 μl of assay buffer to each tube. To validate each assay kit, we created a male and a female pool using extracts from 8 biopsied and 4 stranded males and from 12 biopsied and 10 stranded females, respectively, and tested for parallelism and accuracy. We used two separate pools of pregnant and non-pregnant females to test the testosterone assay for parallelism, but due to limited extracts volume, we combined the two pools for the accuracy test. The parallelism test evaluates whether the antibody from the assay can reliably bind to the targeted hormone and determines the dilution at 50% binding; the accuracy check evaluates how precisely the measured concentrations correlate with the added concentrations of each hormone. Briefly, we serially diluted (1:1, 1:2, 1:4, 1:8 and 1:16) the pool of extracts and tested for parallelism to the standard curve of each assay. Each standard curve was made of 7 points fitting a four parameters logistic curve (4PLC): for testosterone, standards concentrations ranged from 40.96 pg/ml to 10,000 pg/ml, whereas for progesterone, from 50 pg/ml to 3,200 pg/ml. To assess parallelism to the testosterone standard curve, we fitted a linear model between 80% and 20% binding of the standard curves and to the dilutions of each pool. For the progesterone assay, we fitted a 4PLC to the assay standards and dilutions of each pool using the R package *“drc”* [64]. We tested for parallelism using a Student’s t-test to statistically assess the difference in the slope parameter from the standard curve and each curve fitted to the extract pools and considered lack of significance evidence of parallelism [65]. For testosterone, the dilution at 50% binding was 1:1 for all three tested pools (males, pregnant and non-pregnant females). For progesterone, the dilutions binding close to 50% were 1:1 for the pool of males extracts and 1:4 for the pool of females extracts. For the accuracy test, we spiked the assay standards for each hormone kit with an equal volume of sample pool. We plotted and tested the recovered against the added hormone mass for linearity We assayed all standards, zero-standards (or total binding, B0) and all samples in duplicate; we corrected raw concentrations data (pg/ml) for dilution factor and blubber mass, and expressed the final value as ng/g.

The intra-assay percent coefficient of variation (CV) was < 10% for both hormones. If any sample had a CV > 10%, we re-diluted it accordingly and re-assayed. We determined inter-assay validation using two internal controls for testosterone and progesterone respectively. CVs for the two controls were 11.1% and 14.3% for the testosterone assay and 6.5% and 11.3% for the progesterone assay.

### Statistical analysis

**Fig 2:**
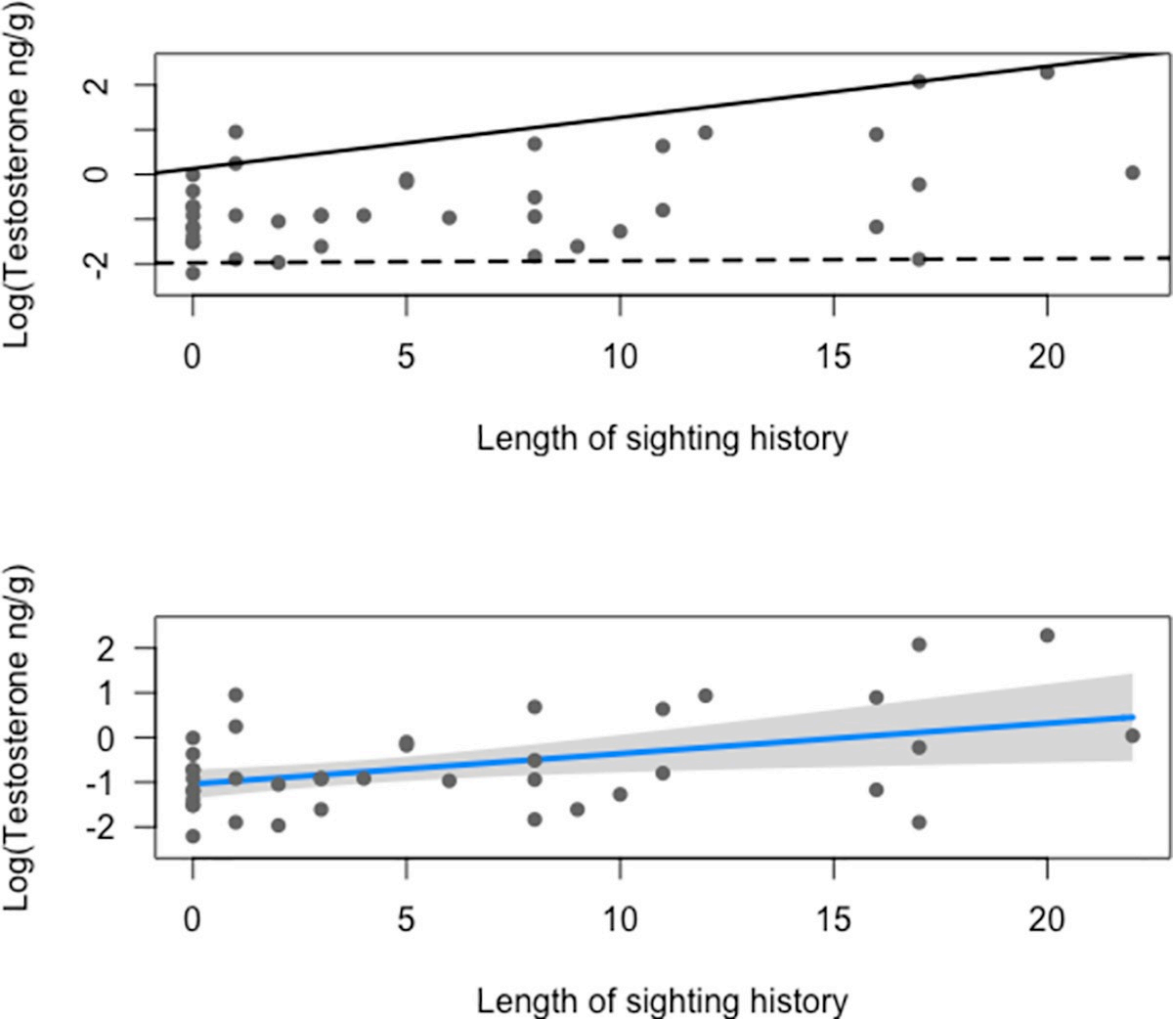
Median and mean log-transformed testosterone concentrations (ng/g) of gray whale males against length of sighting history (LSH). *Top graph*: quantile regression indicated significant increase in the median testosterone concentration in the 95^th^ percentile (*P* < 0.001; solid line), but not in the 5^th^ percentile (*P =* 0.9; dashed line); the analysis indicates that the highest levels of this hormone increase with age, but the lower concentrations do not. *Bottom graph*: linear regression with unequal variance indicated a significant increase of mean testosterone concentrations in response to LSH (*P* = 0.03).

We tested testosterone and progesterone concentrations for normality and homogeneity of variances using Shapiro-Wilk and Bartlett’s tests (Package: *stats* [66]) as part of exploratory data analysis. Based on these results, we log-transformed testosterone and progesterone concentrations to meet statistical requirements of independence and normality. We evaluated the relationship between hormone concentrations and extracted blubber mass using Pearson correlation (Package: *stats* [66]) coefficients with alpha level of 0.05 for statistical significance. All figures but one (Fig 2) showed hormone concentrations back-transformed from the log-scale. We conducted all statistical and graphical analyses using the software R v. 4.0.4 [66].

In order to test testosterone concentrations in response to age, we applied quantile regression and linear regression using a generalized least square approach to log-transformed testosterone concentrations from a subset of males that could be classified either as calf (*n* = 4), immature (*n* = 6) or adult (*n* = 16), using the function “rq” (Package: *quantreg* [67]) and “gls” (Package: *nlme* [68]). The quantile regression tested the relationship between the 5^th^ percentile, the median and the 95^th^ percentile of testosterone and LSH, whereas the linear regression tested for an increase in mean testosterone with LSH, while accounting for an increase in variance with the mean. We analyzed testosterone concentrations in adult males (*n* = 16) for seasonal differences using a two-tailed Student’s test (Package: *stats* [66]) with equal variance by season (summer and fall), and in females for differences among reproductive states using an ANOVA test, followed by a Tukey post-hoc test.

To determine any significant difference in progesterone concentrations among whales of known sex (male and female), age class (calf, immature, adult) and reproductive states (calf, immature, lactating and pregnant), we used an ANOVA test assuming unequal variances followed by pairwise t-test. We then estimated the probability of being pregnant for each non-calf female of known and unknown reproductive status. We used the Expectation-Maximization (EM) algorithm (Package: *mixtools* [69]) to fit two normal distributions to log-transformed progesterone concentrations from all non-calf females, with the assumption that at least two groups would be identified: one cluster of low (non-pregnant) and one of high (pregnant animals) progesterone. We calculated the probability of pregnancy at each progesterone concentration as the ratio of the probability density for the high progesterone group to the sum of the two probability densities, assuming that the normal distribution with the larger mean corresponds to the distribution of progesterone concentrations for pregnant females. We then developed a bootstrap approach, similar to the one applied for humpback whales in Pallin et al. [27] and for blue whales in Melica et al. [54] to quantify the 95% confidence interval around each probability estimate, where progesterone concentrations were re-sampled with replacement 10,000 times. Given the small number of individuals in the "high-progesterone" group we included the four known pregnant females in each bootstrap sample. The probability of pregnancy was estimated for each bootstrap sample and only realistic bootstrap samples resulting in probabilities that asymptotically approached 1 at high progesterone concentrations (90% of total bootstrap samples) were retained. Finally, the 2.5^th^ and 97.5^th^ percentiles of the bootstrapped probabilities at each progesterone concentration were used to construct the 95% confidence band for the estimated probability of pregnancy. We estimated the probability with 95% confidence intervals (CI) for each individual of unknown reproductive status.

For the repeated samples (*n* = 13, five unique males and eight unique females), we analyzed log-transformed hormone concentrations in response to age class and season, using an ANOVA test with correction for repeated samples.

## Results

### Analytical validation

The testosterone assay validated for males, passing the parallelism (*P =* 0.3) and accuracy (*y* = 1.2x-44.5; *R*^*2*^ = 0.99) tests. For females with serial dilutions from both pools (pregnant and non-pregnant), the testosterone assay displayed curves parallel to the standard curve (*P =* 0.1 and 0.2). The combined female pool (both pregnant and non-pregnant) was tested for accuracy and the spiked standards showed linear relationships with the standards *(y* = 1.2x-186.3; *R*^*2*^ = 0.98).

The progesterone assay validated for both females and males with serial dilutions exhibiting parallel displacement to the standard curve (*P* = 0.2 and *P* = 0.6, respectively) and the spiked standards showing linear relationships with the added standard mass (female: *y* = 1.0x-20.6; *R*^*2*^ = 0.99 and male: *y* = 1.0x-63.6; *R* ^*2*^ = 0.99).

### Testosterone

We measured testosterone concentrations in blubber of 40 male gray whales with a minimum age ranging from <1 year old to 22 years of LSH. Concentrations ranged from 0.1 to 9.8 ng/g and the mean (range) for each age class are reported in Table 1. We found no significant correlation between testosterone concentrations and mass of blubber extracted (*r* = 0.2, *P* = 0.2).

The quantile regression showed that there was a significant increase in the 95^th^ percentile of testosterone concentrations (*P*< 0.001), but not in the 5^th^ percentile (*P* = 0.9), implying that the highest levels of this hormone increase with age, but the lower concentrations do not (Fig 2). The overall increase in median testosterone concentrations with LSH was not significant, but the linear regression assuming unequal variance indicated a significant increase in mean testosterone concentrations with LSH (*P* = 0.03) (Fig 2).

**Fig 3:**
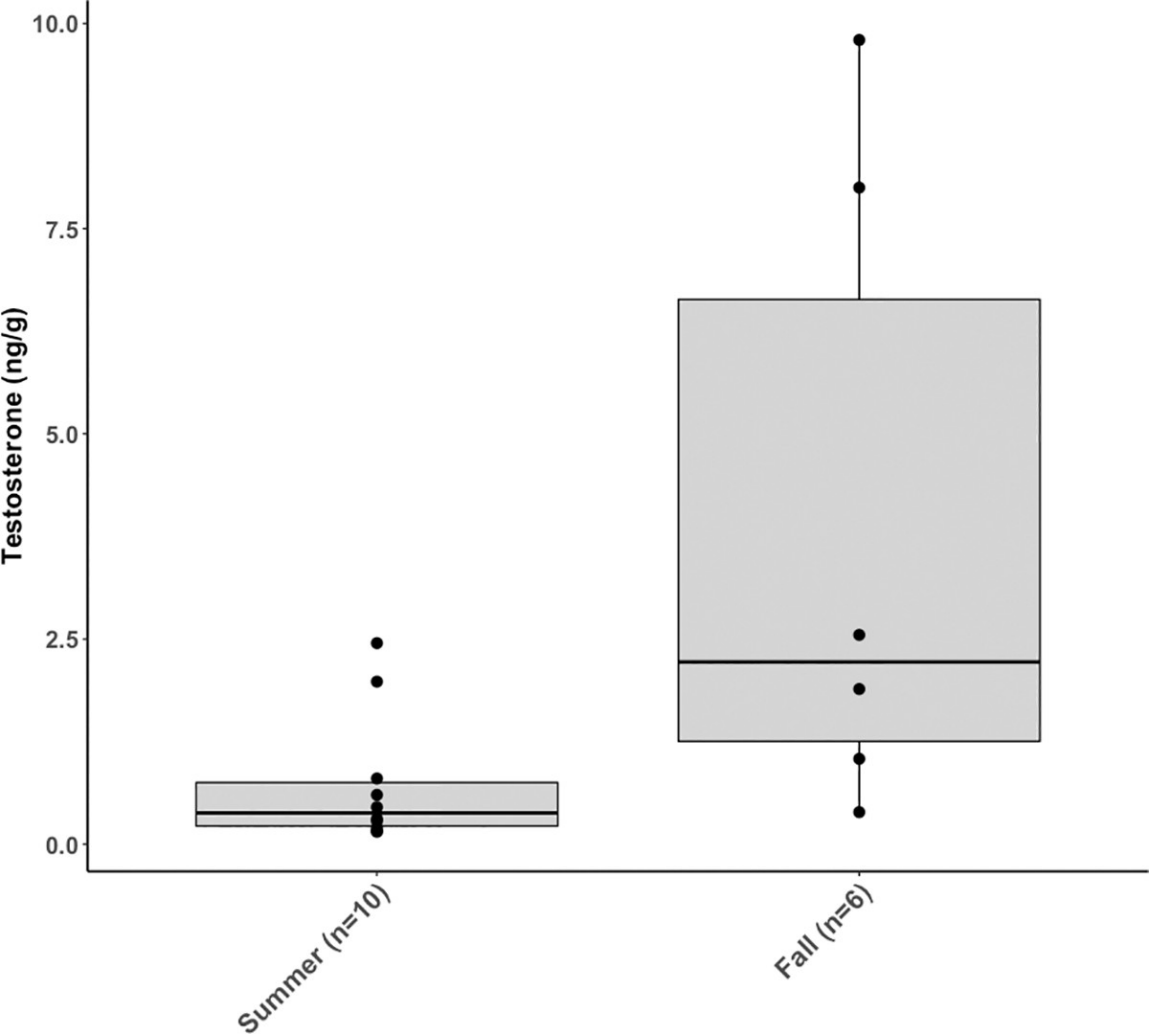
Mean testosterone concentrations (ng/g) were significantly higher in adult males sampled during the fall than during the summer (*P*< 0.05). Boxplots denote median (thick line), upper (75%) and lower (25%) quartile (boxes) and largest and smallest value within 1.5 times interquartile range below 25% and above 75% (whiskers). Outside values are shown as filled circles.

Testosterone concentrations in adult whales showed high variability, ranging from a minimum of 0.1 ng/g to a maximum of 9.8 ng/g. Adult males had a significantly higher mean testosterone concentration in the fall (3.9 ng/g, *n* = 6) than in the summer (0.7 ng/g, *n* = 10) (*t* = -2.9, *df* = 14, *P* = 0.01) (Fig 3).

**Fig 4:**
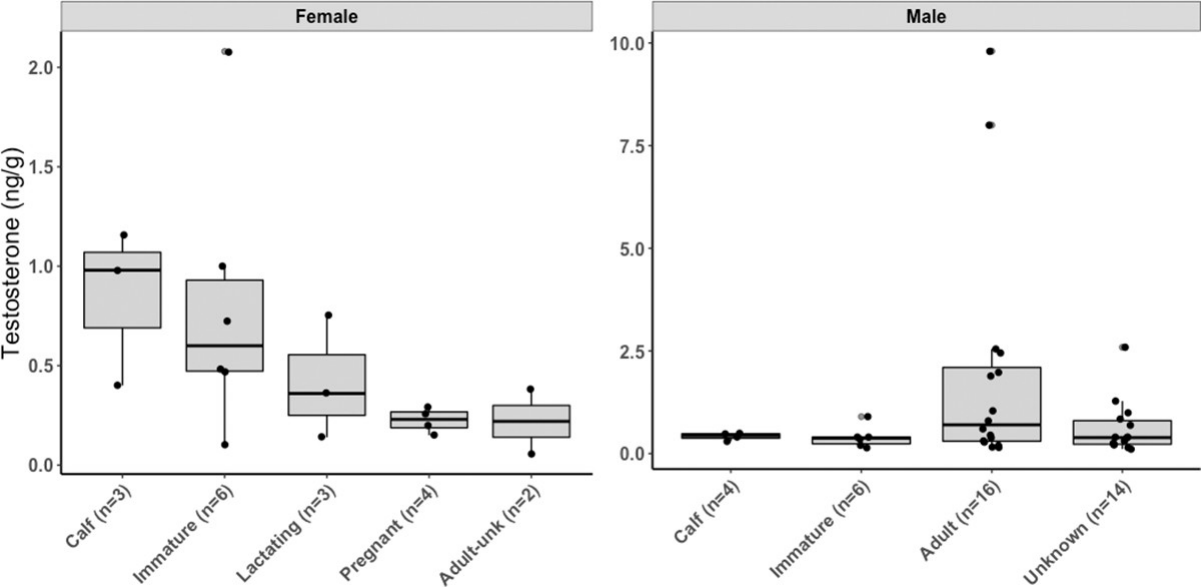
Testosterone concentrations (ng/g) in gray whale of different reproductive states (females) and age class (males). In females, testosterone concentrations were not statistically different among reproductive groups (ANOVA: *F* = 1.9, *df* = 4, *P* = 0.2). Boxplots denote median (thick line), upper (75%) and lower (25%) quartile (boxes) and largest and smallest value within 1.5 times interquartile range below 25% and above 75% (whiskers). Outside values are shown as filled circles.

We detected and measured testosterone concentrations in 18 females from the pregnant (*n* = 4), lactating (*n* = 3), immature (*n* = 6), calf (*n* = 3) and adult unknown (*n* = 2) groups (Table 2 and Fig 4). There was no statistical difference in testosterone concentrations among reproductive groups (ANOVA: *F* = 1.9, *df* = 4, *P* = 0.2).

### Progesterone

**Fig 5:**
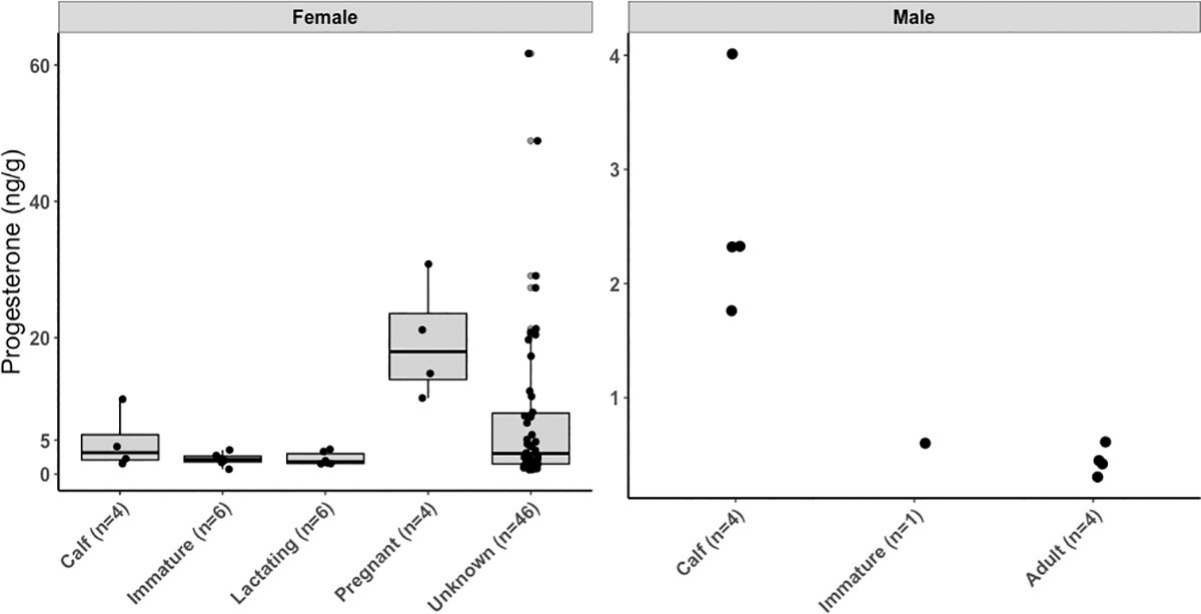
Progesterone concentrations in female and male gray whales. Differences in concentrations were statistically different among reproductive groups for female and male gray whales (ANOVA: *F* = 31.09, *df* = 6.00, *P*< 0.001) of known reproductive status. Boxplots denote median (thick line), upper (75%) and lower (25) quartile (boxes) and largest and smallest value within 1.5 times interquartile range below 25% and above 75% (whiskers).

We measured progesterone concentrations in a total of 66 females, with minimum age from young of the year to 32 years of LSH and concentrations ranged from 0.6 ng/g to 61.7 ng/g (Table 2). We also detected and measured progesterone concentrations in nine individual males, of which four were categorized as calves, one as immature, and four as adult (Table 1). We found no significant correlation between progesterone concentrations and mass of blubber extracted (*r* = 0.09, *P* = 0.4). The comprehensive dataset with females and males of known status was analyzed with an ANOVA test assuming unequal variances followed by a pairwise t-test that indicated concentrations of progesterone to be significantly different among groups (ANOVA: *F* = 34.6, *df* = 5.00, *P*< 0.001). Specifically, we found that females in the pregnant group had significantly higher concentrations than whales from the lactating *(P*< 0.001), immature (*P*< 0.001) and calf (*P* = 0.04) groups (Fig 5), but progesterone concentrations did not vary significantly among females from non-pregnant groups (lactating, immature and calf). Our analysis also indicated that progesterone concentrations were significantly lower in adult males than in any female reproductive groups (pregnant *P*< 0.001; lactating *P*< 0.001; immature *P* = 0.001 and calf *P* = 0.02) and in male calves (*P*< 0.001) (Fig 5).

**Fig 6:**
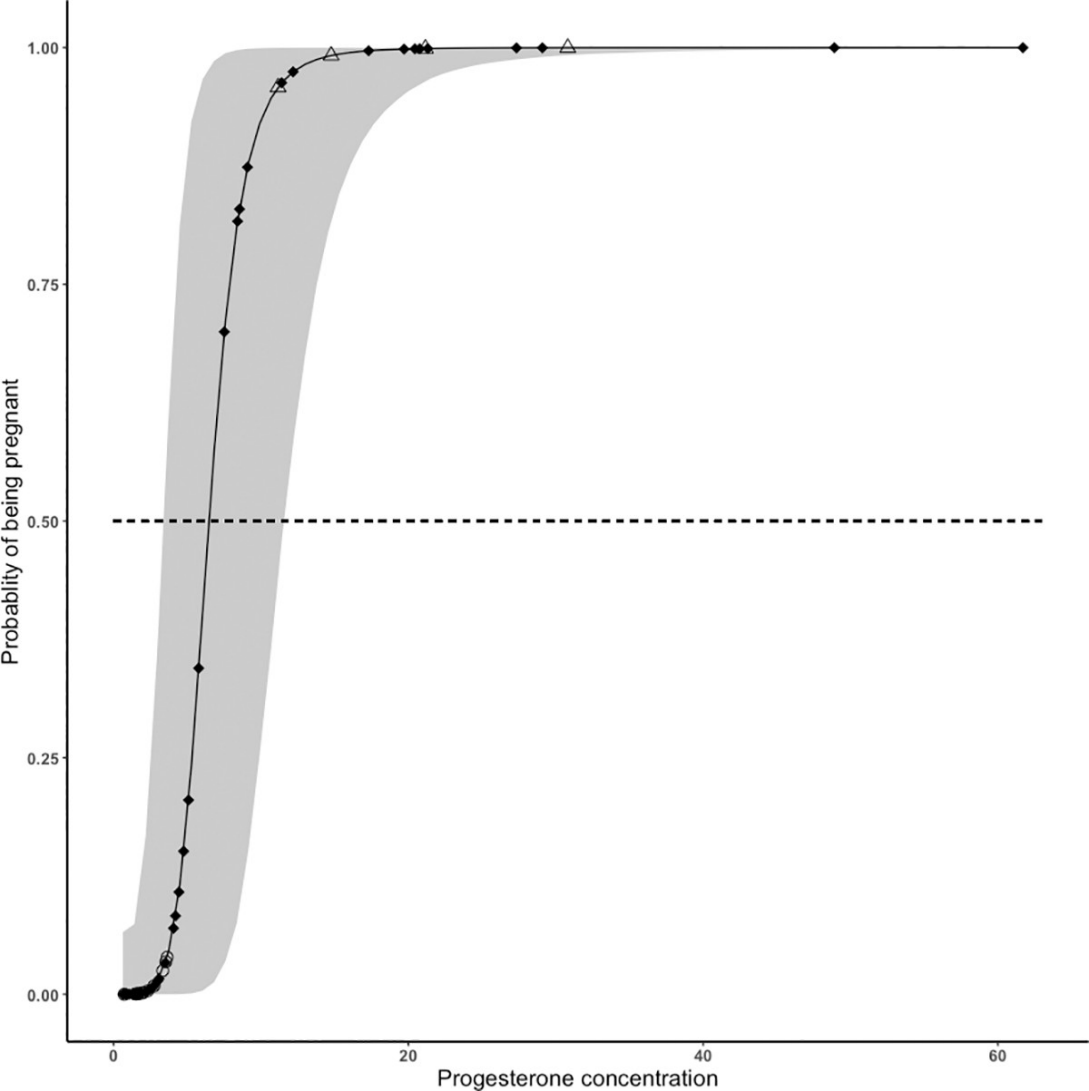
Probability of being pregnant based on progesterone concentrations for all non-calf females, with 95% confidence band calculated using a bootstrapping approach. Shapes of points indicate whales confirmed non-pregnant (immature and lactating; empty circle), pregnant (empty triangle) and unknown (age class unknown and adult; black diamond).

The mixture model suggested that progesterone concentrations from all non-calf females could be best described as a mixture of two normal distributions: the first distribution identified a cluster of whales with low progesterone concentration (mean = 2.0 ng/g (95% CI: 0.6–6.3 ng/g) and included all whales known to be non-pregnant (e.g., lactating and immature) and the second a cluster of whales with high progesterone concentration (mean = 16.9 ng/g (95% CI: 4.8–59.6 ng/g), and included all females confirmed pregnant (Fig 6). The areas of the curves had a 4.5% overlap. Based on these distributions, the model identified 50% probability of being pregnant corresponding to a progesterone concentration of 6.5 ng/g and we calculated the probability of pregnancy for all non-calf females (Table 4). The model found corroboration in the probabilities of pregnancy estimated for whales of known reproductive status: all known pregnant females (*n* = 4) had an estimated probability of being pregnant of 100%, while the probability for all known non-pregnant whales (e.g., lactating and immature; *n* = 12) was lower than 5% (Table 4). The probability of being pregnant ranged from less than 0.01% to 100% for adult females of unknown reproductive status (*n =* 26) and for whales of unknown age class and reproductive status (*n* = 20) (Table 4). Probabilities of being pregnant showed high uncertainty (i.e., broad 95% CI) in whales with intermediate progesterone concentrations (between 5 and 10 ng/g).

**Table 4:** Percent probability of being pregnant assigned to each female gray whale of known and unknown reproductive status. For each whale the probability was calculated based on the progesterone concentration (ng/g), using the two distributions identified via mixture model. Additional information included is feeding group, age class, the year and month of sampling and the year of subsequent sighting including whether with a calf.

CRC ID	Feeding group	Age class	Reproductive status	Progesterone ng/g	Percent probability of being pregnant	Year of sampling	Month of sampling	Year of resighting
860	PCFG	Immature	Immature	3.5	3.3	2004	September	2005
1512	PCFG	Immature	Immature	2	0.2	2013	October	2014
1559	PCFG	Immature	Immature	2.3	0.3	2015	October	2016
1622	PCFG	Immature	Immature	0.7	<0.01	2015	October	2016
1736	PCFG	Immature	Immature	2.7	0.8	2015	October	2016
1822	PCFG	Immature	Immature	1.7	<0.1	2015	October	2016
67	PCFG	Adult	Lactating	3.6	3.9	2004	August	2005
178	PCFG	Adult	Lactating	3.3	2.5	2013	July	2014
372	PCFG	Adult	Lactating	1.7	<0.1	2015	August	2016
525	PCFG	Adult	Lactating	2.0	0.15	2015	October	NA
719	PCFG	Adult	Lactating	1.5	<0.1	2015	October	2016
827	PCFG	Adult	Lactating	1.5	<0.1	2015	September	2018
92	PCFG	Adult	Pregnant	14.8	99.1	2012	August	2013 (with calf)
193	PCFG	Adult	Pregnant	21.2	99.9	2015	October	2016 (with calf)
196	PCFG	Adult	Pregnant	11.2	95.8	2015	October	2016 (with calf)
280	PCFG	Adult	Pregnant	30.8	99.9	2015	October	2016 (with calf)
30	PCFG	Adult	Unknown	17.3	99.7 (95–100)	2015	October	2016
94	PCFG	Adult	Unknown	1.5	<0.1 (7.4x10^-8^–3.1)	2010	October	2011
127	PCFG	Adult	Unknown	0.7	<0.01 (1.4x10-13–1.3)	2010	September	2011
141	PCFG	Adult	Unknown	9.1	87.4 (40–99.8)	2005	July	2006
143	PCFG	Adult	Unknown	7.5	68.4 (13–99)	2015	October	2016
192	PCFG	Adult	Unknown	20.8	99.9 (97–100)	2011	August	2012
204	PCFG	Adult	Unknown	0.8	<0.01 (5.1x10-12–1.3)	2010	July	2011
231	PCFG	Adult	Unknown	3.5	3.3 (8.9x10-3–41.9)	2014	September	2017
238	PCFG	Adult	Unknown	1.3	< 0.1 (0.3x10-7–2.5)	2015	October	2017
242	PCFG	Adult	Unknown	27.3	100 (99.2–100)	2015	September	2018
302	PCFG	Adult	Unknown	0.9	<0.01 (4.6x10-11–1.5)	2010	September	2011
396	PCFG-NPS	Adult	Unknown	0.9	<0.01 (2.6x10-11–1.4)	2010	September	2011
531	NPS	Adult	Unknown	20.4	99.8 (97.8–100)	2016	March	2018
532	PCFG	Adult	Unknown	5.8	34.4 (1.8–93.8)	2012	July	2013
554	PCFG	Adult	Unknown	4.4	10.8 (0.1–71.6)	2015	October	2016
629	PCFG	Adult	Unknown	1.9	0.1 (2.3x10-6–5.7)	2015	October	NA
637	PCFG	Adult	Unknown	48.9	100.0 (99.8–100)	2013	August	2018
657	PCFG	Adult	Unknown	1.2	<0.1 (4.9x10-9–2.2)	2015	October	2016
659	PCFG	Adult	Unknown	1.8	0.1 (1.5x10-6–5.3)	2012	July	2014
668	PCFG	Adult	Unknown	2.7	0.7 (2.8x10-4–17.3)	2012	July	2014
698	PCFG	Adult	Unknown	8.5	82.9 (30–99.7)	2015	September	2016
759	PCFG	Adult	Unknown	2.2	0.3 (2.7x10-5–9.5)	2015	October	NA
760	PCFG	Adult	Unknown	3.0	1.4 (3.5x10-4–18.4)	2015	September	NA
872	PCFG	Adult	Unknown	4.8	15.1 (0.2–80.2)	2013	August	2015
900	PCFG	Adult	Unknown	11.4	96.2 (73–99.9)	2015	October	2018
1067	PCFG	Adult	Unknown	1.6	< 0.1 (1.6x10-7–3.5)	2015	September	NA
826	PCFG	Unknown	Unknown	0.8	<0.01 (6.3x10-12–1.3)	2010	September	2016
842	PCFG	Unknown	Unknown	2.7	0.8 (3.5x10-4–18.4)	2004	September	2005
1053	PCFG	Unknown	Unknown	19.7	99.8 (97.5–100)	2008	October	2012
1059	PCFG	Unknown	Unknown	61.7	100.0 (99.8–100)	2008	October	2009
1118	PCFG	Unknown	Unknown	3.1	1.6 (1.8x10-3–27.8)	2015	September	2016
1172	PCFG	Unknown	Unknown	2.2	0.3 (2.0x10-5–8.8)	2012	August	2013
1201	PCFG	Unknown	Unknown	0.6	<0.01 (4.7x10-14–1.3)	2012	July	2013
1551	PCFG	Unknown	Unknown	1.7	< 0.1 (7.2x10-7–4.5)	2012	August	2013
1597	ENP	Unknown	Unknown	2.5	0.5 (9.5x10-5–13)	2013	October	NA
1598	ENP	Unknown	Unknown	29.1	100 (99.5–100)	2013	October	2014
1600	ENP	Unknown	Unknown	12.2	97.5 (80.8–99.9)	2013	September	NA
1602	ENP	Unknown	Unknown	8.4	81.7 (29.4–99.6)	2013	September	NA
1646	PCFG	Unknown	Unknown	1.7	< 0.1 (5.1x10^-7^–4.2)	2015	October	NA
1681	PCFG	Unknown	Unknown	21.3	99.9 (98.3–99.9)	2014	September	NA
1868	PCFG	Unknown	Unknown	1.1	< 0.01 (4.4x10-10–1.7)	2015	September	2016
1870	ENP	Unknown	Unknown	5.1	20.5 (0.5–86.3)	2015	July	NA
1872	ENP	Unknown	Unknown	4.1	7.0 (0.04–60)	2015	August	NA
1881	ENP	Unknown	Unknown	4.2	8.3 (0.06–65)	2015	August	NA
1890	ENP	Unknown	Unknown	1.3	< 0.1 (0.1x10^-7^–2.5)	2015	September	NA
1899	ENP	Unknown	Unknown	1.4	< 0.1 (0.4x10^-7^–2.9)	2015	October	NA

PCFG, Pacific Coast Feeding Group; NPS, Northern Puget Sound; ENP, Eastern North Pacific; NA, no resighting data available.

### Repeated samples

A total of eight females and five males were sampled twice, in summer and in fall, although not necessarily in the same year. The repeated samples comprised of different age classes and reproductive status (Table 3). In females, the mean progesterone concentration was 3.7 (range 0.2–14.8) ng/g for samples collected in the summer months and 6.8 (range 0.7–19.7) ng/g for those collected in the fall. We found no significant differences in progesterone concentrations between seasons (ANOVA: *F* = 1.1, *df* = 1, *P =* 0.3), age class (*F* = 0.1, *df* = 2, *P =* 0.9) or the combination of both (*F* = 0.4, *df* = 2, *P =* 0.7). In males, the mean (range) testosterone concentrations were 0.4 (0.2–1.0) ng/g for samples collected in the summer and 2.9 (0.2–9.9) ng/g for those collected in the fall (Table 3). We found no statistical difference in testosterone concentrations in response to age class (*F* = 1.2, *df* = 2 *P =* 0.4), season (*F* = 4.5, *df* = 1, *P =* 0.1) and the combination of both (*F* = 3.4, *df* = 2, *P =* 0.1).

## Discussion

The present study validated and measured sex steroids using EIA techniques in 119 blubber samples of gray whales in order to improve the knowledge on the reproductive endocrine processes in this species. Our results indicated that testosterone concentrations in males increased with age until the animals reached maturity and then they varied by season. Specifically, adult males sampled in the fall had higher blubber testosterone concentrations compared to animals sampled in the summer, suggesting physiological preparation for reproduction. In our female dataset, we confirmed progesterone concentrations as a biomarker for pregnancy and developed an analytical model for estimating the probability of being pregnant for female whales of unknown reproductive status. Results from our study complement previous studies that validated and measured steroid hormones in baleen and fecal tissue using EIA [33, 34] and in blubber using nanospray Liquid Chromatography/tandem Mass Spectrometry (nanoLC/MS/MS) [32].

### Testosterone

In the present work, our results indicated that mean testosterone concentrations in male gray whales generally increased with age, indicating that the development of male sexual characteristics is a function of age. This result is consistent with other studies: for example, Rice and Wolman [19] examined testes from immature and adult male gray whales and observed spermatogenesis in seminiferous tubules from adult males. They also indicated open and wider seminiferous tubules as well as bigger testes weights as indicators of sexual maturity and onset of mating. Specifically, diameters of seminiferous tubules were higher in mature animals during the southbound migration [19]. In other mysticete species, lower concentrations of testosterone have been observed in immature compared to mature humpback and fin whales [41, 70], with increased variability observed mainly in adult animals suggesting that other variables (e.g., season) likely affect concentrations of this hormone. Similarly, when we analyzed testosterone concentrations using quantile regression, we found that only elevated concentrations increased significantly with age (Fig 2), whereas lower concentrations did not. This means that in older males, variability in testosterone concentrations is broader, hinting that there might be other factors (e.g., season) affecting hormone levels in adult males.

Cyclicity or seasonal trends in testosterone concentrations from a variety of tissue types have been reported for humpback, blue, fin, and North Atlantic right whales [31, 41, 54, 59, 60, 70], with elevated concentrations in the months approaching the breeding season, indicating physiological preparation to mate. The present study found blubber testosterone concentrations had higher variability in adult males (range 0.1–9.8 ng/g), leading to the hypothesis of a seasonal trend. Statistical analysis indicated testosterone concentrations to be significantly higher in animals sampled in the fall compared to samples collected in the summer (Fig 3), supporting seasonality as the main explanatory factor. All adult males analyzed for seasonal changes in testosterone were identified as part of the PCFG, and sampled between June and October, while on their feeding grounds. Increased testosterone concentrations over time likely indicate preparation for mating through spermatogenesis as the timing of migration approaches. Repeated samples from two adult males support these results, as both animals had at least 10 times higher testosterone concentrations in blubber collected in the fall (Table 3) compared to their respective samples from the summer. Increased sample size and analysis of samples collected between December and May, while the animals are in their northward and southward migratory flow, is necessary to have a more complete understanding of the testosterone annual cycle.

We validated and measured testosterone concentrations in female gray whales, and when compared across reproductive states we found that immature females had generally high concentrations when compared to mature females. Among adult females, lactating whales had testosterone concentrations twice as high compared to pregnant animals, although these differences were not statistically significant (Fig 4). Elevated androgens (i.e., testosterone) were found in blubber of female humpback whales close to parturition [71] and in feces of pregnant and lactating North Atlantic right whales [61]. The pregnant females in the present study were all sampled at least two months before the estimated parturition date (late December–early January [19]). Thus, their blubber testosterone might not reflect a spike in androgens occurring in late gestational phase. On the other hand, elevated blubber testosterone in lactating females may be the delayed result of such surge, whereas in calves the consequence of maternal transfer in milk. In preparation for lactation, pregnant gray whales increase their weight during the feeding season 25–30% more than whales in other reproductive states [5, 19]. Further, the milk of gray whales has the highest fat content (53%) among cetaceans [72, 73]. During migration and lactation, the accumulated body fat and blubber are used as energy sources and transferred to the calf. Maternal offloading of contaminants and trace metals has been documented for this species [42] and other whales (e.g., fin whales [74]), suggesting that lipophilic steroid hormones are likely also transferred.

### Progesterone

Our study provides evidence that progesterone can be used as an indicator of pregnancy, as significantly elevated concentrations were found in females confirmed as pregnant (Fig 5) compared to other reproductive states. In addition, we developed a model that used progesterone concentrations from non-calf females to calculate the probability of being pregnant and their 95% confidence intervals (Table 4). The model indicated that female whales with known reproductive status, that is known pregnant whales, had an estimated probability of being pregnant higher than 95%, whereas known non-pregnant females (e.g., lactating and immature whales) had less than 5% estimated probability of being pregnant. Using these whales as references it is reasonable to conclude that all whales with probability higher than 95% were likely pregnant, while all whales with probability lower than 5% were likely non-pregnant at the time of sampling. With the thresholds developed from these probabilities, we hypothesized that out of 46 whales of unknown reproductive status (both in the adult and unknown age class), 11 were likely pregnant and 25 likely non-pregnant. The remaining ten individuals had mid-range progesterone concentrations and resulting intermediate probabilities with high levels of uncertainty (expressed as 95% confidence band; Fig 6), thus they could not be assigned a reproductive status accurately.

Of the likely pregnant whales, four were sighted the year after sampling not accompanied by a calf in late summer or fall. It is possible that these females had calves which had already been weaned by the time of their sighting in the subsequent year; however, whales in the lactating group were sampled between July and October, and whales in the calves group between June and November, suggesting that calves tend to stay close to their mothers on the feeding grounds [19, 75].

A high probability of pregnancy but no sighted calves the subsequent year might also be a result of reproductive failure, through either the loss of a calf post-parturition or the loss of the fetus, also referred to as a spontaneous abortion [76, 77]. Calf mortality in gray whales has been reported to be high. Specifically, Swartz and Jones [78] suggested a 31% decrease in the number of calves between those counted in the Mexican lagoons and the calves counted migrating past Central California with their mothers. Accordingly, 60% of calf mortality was estimated to occur south of 49°N [79]. Finally, it is possible that some of the 11 likely pregnant females were primiparous (i.e., in their first pregnancy). Calves of primiparous females in marine mammals often have lower survival than calves born to multiparous (i.e., that had given birth at least once) females. For example, multiparous bottlenose dolphins showed higher calves survival [80] and older Antarctic fur seals *(Arctocephalus gazella)* had greater reproductive performance than younger ones [81]. Based on the CRC catalog, only one of these females was sighted with a calf before the sampling occurred (CRC 242), so it is possible that despite a LSH longer than 8 years, these whales might have been in their first pregnancy. Furthermore, we were not able to assign five of these females to the adult age class, as they had a limited LSH (S1 Table), thus they could have been young individuals.

Elevated progesterone may also be the result of ovulation; however, few studies have reported that endocrine biomarkers for ovulation can be detected in blubber tissue [82]. In minke whales, no significant difference was found between blubber progesterone in ovulating and pregnant females [82]. Other hormones, such as estrogens or luteinizing hormone may be more informative of ovulation [83–85], but likely because of their pulsatile action they are more easily detectable in serum, urine [84] or feces [61].

Ten whales had an estimated probability of being pregnant between 6% and 90% and broad confidence intervals in most cases. Medium to high progesterone concentrations may reflect different stages of the ovulatory cycle. Rice and Wolman [19] estimated that females ovulate between late November and early December, with potentially later ovulations if there was a failure in conception. The samples mentioned above were collected between the months of July and October, indicating their progesterone concentrations are unlikely to be a reflection of ovulation.

Pseudopregnancy could be an alternative explanation for mid to high progesterone concentrations. It consists of the retention of a corpus luteum, despite the lack of conception, for an amount of time longer than normal [37]. Pseudopregnancy is known to occur in many odontocetes species, often in a recurring pattern [85], but there is no evidence for baleen whales.

More than half of the whales with unknown reproductive status had a probability of being pregnant lower than 5% (25 out of 46), with about half sighted the year after sampling not accompanied by a calf. Of the remaining, three were sighted two years after the year of sampling, one 6 years later and eight had no resightings recorded. Low progesterone concentrations and absence of calf might be indicative of females in a resting status or that have not reached sexual maturity. Fecal progesterone concentrations in resting whales were similar to those categorized as lactating, for blue whales [30] and North Atlantic right whales [61]. In feces from PCFG gray whales categorized as resting, the mean progesterone concentration was similar to the mean for immature females, and about half the mean concentration for pregnant females [34]; however, in Lemos et al [34], the range of concentrations in resting females appears to be pretty broad and overlaps with that of pregnant females. Furthermore, no significant difference in blubber progesterone concentrations was found between resting, ovulating and pregnant minke whales [82]. The same study, however, found significantly lower progesterone concentrations in immature whales, indicating this biomarker can be used to differentiate between immature and mature females for that species [82].

In the present study, the applied age of sexual maturity is based on Rice and Wolman [19], which estimated mean age of sexual maturity at 8 years old (with a range from 5 to 11 years) based on earplug growth layers and gonads; however, it is possible that this parameter has changed over the past 50 years and it requires reanalysis and clarification [20], especially if applied to a distinct feeding group such as the PCFG. Age of sexual maturity is density-dependent [86], and it is assumed to increase in high-density populations [87]. Both the ENP gray whale population and the PCFG have increased over the last several decades [8, 88], and it is possible that the age of sexual maturity has also increased. Updated estimates of this parameter are necessary for more precise and accurate analyses of whales in different reproductive states.

Despite the complexity in clearly categorizing each individual, it is noteworthy that the female dataset in the present study reflects multiple reproductive states, with progesterone clusters not limited to high and low classifications. The mixture model applied in this study indicated a 4.5% overlap in the two distributions, confirmed also by overlapping of confidence intervals. This is a somewhat bigger overlap than observed in similar studies in which a gap or minimal overlap was found between groups with low and high concentrations [27, 31]. However, results from work presented here confirmed that progesterone can be used as an indicator of pregnancy, as demonstrated for other mysticete species [25, 29, 31, 38, 39, 54]. While absolute concentrations of hormones should not be compared unless proper interlaboratory calibrations have been conducted, it is worth noticing that the mean progesterone concentration (19.5 ng/g; Table 1) in blubber of pregnant gray whales from the present study is low compared to other species, sampled in their winter or summer grounds. For instance, although the studies utilized different EIA kits, Melica et al. [54] reported a mean progesterone concentration of 81.4 ng/g in blubber of pregnant blue whales sampled in both winter and summer grounds, whereas Atkinson et al. [29] reported a mean of 40.3 ng/g from biopsies of pregnant blue whales in their winter grounds. A similar disparity is seen in feces: fecal samples from pregnant gray whale sampled in their summer grounds had mean progestins metabolites of 157.4 ng/g [34], whereas in blue whales feces collected while the whales were in their wintering grounds, mean progesterone was 1292.6 ng/g [30]. However, these studies were conducted in different laboratories and used different EIA kits. In humpback whales, a species more comparable in size to gray whales, calculated blubber progesterone thresholds for pregnancy are quite variable [71], ranging from 19.3 ng/g [27, 28] to 55.0 ng/g [27], with data from different laboratories. Progesterone accumulation in blubber is likely affected by physio-morphological aspects (e.g., the species average size, blubber depth) and the time of sampling, likely reflecting different gestational stages.

We also validated and measured progesterone concentrations in males (Fig 5). Our results found that adult males had significantly lower progesterone concentrations than females from any reproductive groups and male calves (Fig 5). Like testosterone, elevated progesterone in calves is likely a result of maternal transfer and this hypothesis was further supported by the fact that progesterone concentrations were not different between male and female calves. Mean calving time for gray whales is the beginning of January and calves are normally weaned 6–7 months post-partum [19], indicating these calves could be 6–11 months old and thus weaned. Progesterone concentrations were more variable in females (range:1.5–11.0 ng/g; *n* = 4) than in males (range: 1.8–4.0 ng/g; *n* = 4) calves. However, the limited sample size did not allow for an accurate comparison of progesterone concentrations over time of year, in order to better understand the turnover of hormone in blubber.

## Conclusions

The present study provides new fundamental information on concentrations of reproductive hormones in blubber of gray whales. The results for male gray whales align with what was found in other species, suggesting a seasonal cycle in testosterone concentrations in adult males to be detectable in blubber tissue [31, 54, 89]. Elevated testosterone concentrations were found in animals sampled between late September and October, likely indicating preparation for mating. Furthermore, because all sampled adult males were part of the PCFG, this study indicates that physiological preparation for reproduction begins while on their feeding grounds. For female gray whales, the present study highlights the complexity of physiological reproductive profiles, and the results presented here are innovative in developing a model to calculate the probability of being pregnant based on progesterone concentrations. With most samples collected from individuals in the PCFG, these data represent a milestone in better understanding reproductive profiles in gray whales from this region, as well as in general for gray whales. Based on these results, a more accurate estimate of key reproductive parameters for gray whales is now possible.

## Supporting information

S1 Table Summary information for each individual whale (*n* = 106). For each individual whale the following data are reported: NOAA SWFSC Marine Mammal and Sea Turtle Research Collection identification number (T_ID), CRC ID, feeding group, sex, length of sighting history, age class, reproductive status, location of sampling, progesterone (ng/g) and testosterone (ng/g) concentrations.
(DOCX)
