# Supplementary material for: Application of endocrine biomarkers to update information on reproductive physiology in gray whale (Eschrichtius robustus)
Source: PLoS One. 2021 Aug 3;16(8):e0255368. doi: 10.1371/journal.pone.0255368 (PMC8330940; doi:10.1371/journal.pone.0255368)
Supplement: S1 Table — For each individual whale the following data are reported: NOAA SWFSC Marine Mammal and Sea Turtle Research Collection identification number (T_ID), CRC ID, feeding group, sex, length of sighting history, age class, reproductive status, location of sampling, progesterone (ng/g) and testosterone (ng/g) concentrations. (DOCX) [file pone.0255368.s001.docx]

**S1 Table. Summary information for each individual whale (*n =* 106).** For each individual whale the following data are reported: NOAA SWFSC Marine Mammal and Sea Turtle Research Collection identification number (T_ID), CRC ID, feeding group, sex, length of sighting history, age class, reproductive status, location of sampling, progesterone (ng/g) and testosterone (ng/g) concentrations.

| **T_ID** | **CRC ID** | **Feeding Group** | **Sex** | **LSH** | **Age Class** | **Reproductive Status** | **Season** | **Area** | **Progesterone ng/g** | **Testosterone ng/g** |
| --- | --- | --- | --- | --- | --- | --- | --- | --- | --- | --- |
| 131784 | 67 | PCFG | female | 12 | Adult | Lactating | Summer | WA | 3.63 |  |
| 131786 | 842 | PCFG | female | 0 | Unknown | Unknown | Fall | WA | 2.72 |  |
| 131787 | 860 | PCFG | female | 1 | Juvenile | Immature | Fall | WA | 3.54 | 2.08 |
| 131782 | 141 | PCFG | female | 15 | Adult | Unknown | Summer | WA | 9.08 |  |
| 135088 | 1059 | PCFG | female | 0 | Unknown | Unknown | Fall | WA | 61.7 |  |
| 135089 | 1053 | PCFG | female | 0 | Unknown | Unknown | Fall | WA | 19.71 |  |
| 135092 | 204 | PCFG | female | 14 | Adult | Unknown | Summer | WA | 0.82 |  |
| 135242 | 396 | NPS | female | 11 | Adult | Unknown | Summer | WA | 0.9 |  |
| 132263 | 127 | PCFG | female | 24 | Adult | Unknown | Fall | CA | 0.67 |  |
| 135260 | 302 | PCFG | female | 12 | Adult | Unknown | Fall | WA | 0.93 |  |
| 135250 | 826 | PCFG | female | 6 | Unknown | Unknown | Fall | WA | 0.83 |  |
| 135267 | 94 | PCFG | female | 17 | Adult | Unknown | Fall | OR | 1.48 |  |
| 148864 | 192 | PCFG | female | 15 | Adult | Unknown | Summer | WA | 20.77 |  |
| 148884 | 1330 | PCFG | female | 0 | Calf | Calf | Fall | WA | 1.54 |  |
| 160171 | 532 | PCFG | female | 12 | Adult | Unknown | Summer | WA | 5.77 |  |
| 160173 | 1201 | PCFG | female | 3 | Unknown | Unknown | Summer | WA | 0.63 |  |
| 160176 | 668 | PCFG | female | 10 | Adult | Unknown | Summer | WA | 2.67 |  |
| 160174 | 659 | PCFG | female | 10 | Adult | Unknown | Summer | WA | 1.82 | 0.38 |
| 160179 | 92 | PCFG | female | 19 | Adult | Pregnant | Summer | WA | 14.76 | 0.26 |
| 160180 | 1172 | PCFG | female | 3 | Unknown | Unknown | Summer | WA | 2.19 |  |
| 160181 | 1551 | PCFG | female | 0 | Unknown | Unknown | Summer | WA | 1.73 |  |
| 164093 | 178 | PCFG | female | 18 | Adult | Lactating | Summer | WA | 3.33 | 0.75 |
| 164097 | 637 | PCFG | female | 12 | Adult | Unknown | Summer | WA | 48.9 |  |
| 164096 | 872 | PCFG | female | 8 | Adult | Unknown | Summer | WA | 4.75 |  |
| 164101 | 1600 | ENP | female | 0 | Unknown | Unknown | Summer | WA | 12.18 |  |
| 164102 | 1602 | ENP | female | 0 | Unknown | Unknown | Summer | WA | 8.41 |  |
| 164104 | 1512 | PCFG | female | 1 | Juvenile | Immature | Fall | WA | 2 | 0.47 |
| 164105 | 1598 | ENP | female | 0 | Unknown | Unknown | Fall | WA | 29.09 |  |
| 164106 | 1597 | ENP | female | 0 | Unknown | Unknown | Fall | WA | 2.46 |  |
| 203265 | 1681 | PCFG | female | 1 | Unknown | Unknown | Spring | WA | 21.31 |  |
| 203272 | 1741 | PCFG | female | 0 | Calf | Calf | Summer | WA | 2.26 | 0.4 |
| 203273 | 1834 | PCFG | female | 0 | Calf | Calf | Summer | WA | 4.04 | 0.98 |
| 203274 | 231 | PCFG | female | 16 | Adult | Unknown | Fall | WA | 3.52 |  |
| 196451 | 1870 | ENP | female | 0 | Unknown | Unknown | Summer | AK | 5.08 |  |
| 203282 | 1838 | PCFG | female | 0 | Calf | Calf | Summer | WA | 10.98 | 1.16 |
| 196454 | 1881 | ENP | female | 0 | Unknown | Unknown | Summer | AK | 4.2 |  |
| 196453 | 1872 | ENP | female | 0 | Unknown | Unknown | Summer | AK | 4.06 |  |
| 203285 | 372 | PCFG | female | 16 | Adult | Lactating | Summer | WA | 1.65 | 0.36 |
| 196459 | 1890 | ENP | female | 0 | Unknown | Unknown | Summer | AK | 1.31 |  |
| 196461 | 760 | PCFG | female | 13 | Adult | Unknown | Summer | AK | 2.99 |  |
| 196479 | 242 | PCFG | female | 17 | Adult | Unknown | Fall | BC | 27.33 | 0.06 |
| 196472 | 698 | PCFG | female | 13 | Adult | Unknown | Fall | BC | 8.54 |  |
| 203289 | 1118 | PCFG | female | 6 | Unknown | Unknown | Fall | WA | 3.08 |  |
| 203287 | 827 | PCFG | female | 11 | Adult | Lactating | Fall | WA | 1.52 |  |
| 196487 | 1067 | PCFG | female | 7 | Adult | Unknown | Fall | BC | 1.56 |  |
| 196473 | 1868 | PCFG | female | 0 | Unknown | Unknown | Fall | BC | 1.06 |  |
| 196492 | 143 | PCFG | female | 19 | Adult | Unknown | Fall | BC | 7.51 |  |
| 196498 | 554 | PCFG | female | 18 | Adult | Unknown | Fall | WA | 4.43 |  |
| 196503 | 1622 | PCFG | female | 2 | Juvenile | Immature | Fall | WA | 0.71 | 0.72 |
| 203292 | 1736 | PCFG | female | 1 | Juvenile | Immature | Fall | WA | 2.74 | 0.48 |
| 196511 | 525 | PCFG | female | 15 | Adult | Lactating | Fall | BC | 1.95 |  |
| 196514 | 30 | PCFG | female | 32 | Adult | Unknown | Fall | BC | 17.3 |  |
| 196518 | 759 | PCFG | female | 17 | Adult | Unknown | Fall | BC | 2.24 |  |
| 196517 | 1899 | ENP | female | 0 | Unknown | Unknown | Fall | BC | 1.43 |  |
| 196521 | 900 | PCFG | female | 11 | Adult | Unknown | Fall | BC | 11.4 |  |
| 196519 | 629 | PCFG | female | 19 | Adult | Unknown | Fall | BC | 1.88 |  |
| 196520 | 238 | PCFG | female | 20 | Adult | Unknown | Fall | BC | 1.32 |  |
| 196526 | 193 | PCFG | female | 21 | Adult | Pregnant | Fall | BC | 21.15 | 0.2 |
| 196529 | 719 | PCFG | female | 13 | Adult | Lactating | Fall | BC | 1.54 | 0.14 |
| 196531 | 280 | PCFG | female | 17 | Adult | Pregnant | Fall | BC | 30.81 | 0.29 |
| 196533 | 1822 | PCFG | female | 1 | Juvenile | Immature | Fall | BC | 1.7 | 1 |
| 196545 | 196 | PCFG | female | 19 | Adult | Pregnant | Fall | BC | 11.16 | 0.15 |
| 196553 | 1646 | PCFG | female | 2 | Unknown | Unknown | Fall | OR | 1.69 |  |
| 196551 | 1559 | PCFG | female | 3 | Juvenile | Immature | Fall | OR | 2.3 | 0.1 |
| 196550 | 657 | PCFG | female | 13 | Adult | Unknown | Fall | OR | 1.24 |  |
| 210306 | 531 | NPS | female | 16 | Adult | Unknown | Spring | WA | 20.44 |  |
| 131783 | 785 | PCFG | male | 1 | Unknown | Male-Unknown | Summer | WA |  | 2.59 |
| 132896 | 89 | PCFG | male | 17 | Adult | Male-Adult | Summer | WA |  | 0.15 |
| 132898 | 823 | PCFG | male | 6 | Unknown | Male-Unknown | Summer | WA |  | 0.38 |
| 132276 | 510 | PCFG | male | 10 | Adult | Male-Adult | Fall | CA |  | 0.28 |
| 135256 | 107 | PCFG | male | 16 | Adult | Male-Adult | Fall | WA | 0.61 | 0.31 |
| 132901 | 720 | PCFG | male | 8 | Adult | Male-Adult | Fall | WA | 0.31 | 0.16 |
| 135265 | 696 | PCFG | male | 8 | Adult | Male-Adult | Fall | OR |  | 0.39 |
| 134496 | 1110 | PCFG | male | 0 | Unknown | Male-Unknown | Fall | WA |  | 0.11 |
| 134497 | 37 | PCFG | male | 22 | Adult | Male-Adult | Fall | WA |  | 1.04 |
| 148851 | 787 | PCFG | male | 8 | Adult | Male-Adult | Summer | WA |  | 1.98 |
| 148857 | 537 | PCFG | male | 11 | Adult | Male-Adult | Summer | WA |  | 0.45 |
| 148859 | 1047 | PCFG | male | 3 | Juvenile | Male-Immature | Summer | WA |  | 0.4 |
| 148870 | 714 | PCFG | male | 9 | Adult | Male-Adult | Fall | WA |  | 0.2 |
| 148872 | 1051 | PCFG | male | 3 | Unknown | Male-Unknown | Fall | WA |  | 0.4 |
| 160183 | 1521 | PCFG | male | 0 | Calf | Male-Calf | Fall | WA | 4.01 | 0.48 |
| 164091 | 1693 | PCFG | male | 0 | Calf | Male-Calf | Summer | WA | 2.33 | 0.49 |
| 164094 | 1603 | ENP | male | 0 | Unknown | Male-Unknown | Summer | WA |  | 0.31 |
| 164099 | 840 | PCFG | male | 8 | Adult | Male-Adult | Summer | WA |  | 0.6 |
| 164098 | 1604 | PCFG | male | 0 | Unknown | Male-Unknown | Summer | WA |  | 0.99 |
| 164100 | 140 | PCFG | male | 16 | Adult | Male-Adult | Summer | WA |  | 2.45 |
| 164103 | 1596 | ENP | male | 0 | Unknown | Male-Unknown | Summer | WA |  | 0.69 |
| 203268 | 1254 | PCFG | male | 4 | Juvenile | Male-Immature | Summer | WA | 0.6 | 0.4 |
| 203270 | 1303 | PCFG | male | 3 | Juvenile | Male-Immature | Summer | WA |  | 0.2 |
| 203271 | 1718 | PCFG | male | 1 | Unknown | Male-Unknown | Summer | WA |  | 0.15 |
| 203281 | 296 | PCFG | male | 17 | Adult | Male-Adult | Summer | WA |  | 0.8 |
| 203284 | 1839 | PCFG | male | 0 | Calf | Male-Calf | Summer | WA | 2.32 | 0.3 |
| 203290 | 226 | PCFG | male | 17 | Adult | Male-Adult | Fall | WA |  | 8 |
| 196482 | 824 | PCFG | male | 11 | Adult | Male-Adult | Fall | WA | 0.42 | 1.89 |
| 203288 | 1864 | PCFG | male | 0 | Calf | Male-Calf | Fall | WA | 1.76 | 0.4 |
| 196491 | 1830 | PCFG | male | 1 | Unknown | Male-Unknown | Fall | BC |  | 0.4 |
| 196490 | 1897 | ENP | male | 0 | Unknown | Male-Unknown | Fall | BC |  | 0.25 |
| 203291 | 166 | PCFG | male | 20 | Adult | Male-Adult | Fall | WA | 0.45 | 9.8 |
| 196504 | 797 | PCFG | male | 12 | Adult | Male-Adult | Fall | WA |  | 2.55 |
| 196494 | 1256 | PCFG | male | 5 | Unknown | Male-Unknown | Fall | WA |  | 0.84 |
| 196501 | 1623 | PCFG | male | 2 | Juvenile | Male-Immature | Fall | WA |  | 0.35 |
| 196502 | 1654 | PCFG | male | 2 | Juvenile | Male-Immature | Fall | WA |  | 0.14 |
| 196497 | 1898 | ENP | male | 0 | Unknown | Male-Unknown | Fall | WA |  | 0.22 |
| 196528 | 1900 | ENP | male | 0 | Unknown | Male-Unknown | Fall | BC |  | 0.22 |
| 196534 | 1739 | PCFG | male | 1 | Unknown | Male-Unknown | Fall | BC |  | 1.28 |
| 196554 | 1234 | PCFG | male | 5 | Juvenile | Male-Immature | Fall | OR |  | 0.9 |

PCFG, Pacific Coast Feeding Group; ENP, Eastern North Pacific; NPS, North Puget Sound;

LSH, length of sighting history

CA, California; OR, Oregon; WA, Washington; AK, Alaska; BC, British Columbia
